# Supramolecular Photothermal Nanomedicine Mediated Distant Tumor Inhibition via PD-1 and TIM-3 Blockage

**DOI:** 10.3389/fchem.2020.00001

**Published:** 2020-02-14

**Authors:** Tong-Yi Huang, Guang-Liang Huang, Chun-Yang Zhang, Bo-Wen Zhuang, Bao-Xian Liu, Li-Ya Su, Jie-Yi Ye, Ming Xu, Ming Kuang, Xiao-Yan Xie

**Affiliations:** ^1^Department of Medical Ultrasonics, Institute of Diagnostic and Interventional Ultrasound, First Affiliated Hospital of Sun Yat-Sen University, Guangzhou, China; ^2^Department of Liver Surgery, First Affiliated Hospital of Sun Yat-Sen University, Guangzhou, China

**Keywords:** liposome, ICG, photothermal therapy, distant tumor, PD-1, TIM-3, immune checkpoint blockade

## Abstract

Supramolecular nanoparticles for photothermal therapy (PTT) have shown promising therapeutic efficacy in the primary tumor and great potential for turning the whole-body immune microenvironment from “cold” to “hot,” which allows for the simultaneous treatment of the primary tumor and the metastatic site. In this work, we develop a liposome-based PTT nanoparticle through the self-assembly of FDA-approved intravenous injectable lipids and a photothermal agent, indocyanine green (ICG). The obtained ICG-liposome shows long-term storage stability, high ICG encapsulation efficiency (>95%), and enhanced near-infrared (NIR) light-triggered photothermal reaction both *in vitro* and *in vivo*. The ICG-liposome efficiently eradicated the primary tumor upon laser irradiation in two colon cancer animal models (CT26 and MC38) and promoted the infiltration of CD8 T cells to distant tumors. However, PTT from ICG-liposome shows only a minimal effect on the inhibition of distant tumor growth in long-term monitoring, predicting other immunosuppressive mechanisms that exist in the distant tumor. By immune-profiling of the tumor microenvironment, we find that the distant tumor growth after PTT highly correlates to compensatory upregulation of immune checkpoint biomarkers, including program death-1 (PD-1), T-cell immunoglobulin, and mucin domain-containing protein 3 (TIM-3), in tumor-infiltrating CD8 T cells. Based on this mechanism, we combine dual PD-1 and TIM-3 blockade with PTT in an MC38 tumor model. This combo successfully clears the primary tumor, generates a systemic immune response, and inhibits the growth of the distant tumor. The ICG-liposome-combined PD-1/TIM-3 blockade strategy sheds light on the future clinical use of supramolecular PTT for cancer immunotherapy.

## Introduction

In clinical practice, the clearance of the primary tumor can be realized through surgical removal, thermal-related ablation, and radiation (Chu and Dupuy, 2014; Lee et al., 2014; Chen et al., 2016a; Yang et al., 2017), while the metastatic site often escapes the locoregional destruction due to the limitation of tumor detection or accessibility. Unsolved metastasis is one of the major causes of tumor recurrence and treatment failure in cancer patients (Mahnken et al., 2013; Sag et al., 2016; Hoffmann, 2017). Compared to traditional surgical removal, ablation and radiation not only help the eradication of the primary tumor but also serve as effective ways to promote tumor-related immune responses, which is also known as the transformation from cold tumor to hot tumor (Finkelstein et al., 2011; Mehta et al., 2016; Tang et al., 2016). As highly focused therapies, ablation and radiation have multiple immunogenic properties, such as inducing immunogenic tumor cell death and initiating tumor antigen presentation, while the host immune system is not compromised (Corso et al., 2011; Rubner et al., 2012; Chu and Dupuy, 2014). Furthermore, they can cause local inflammation mediated by immediate neutrophil infiltration, which can make the cold tumor hot by initiating tumor infiltrating lymphocytes (TILs). The improvement to tumor immune microenvironment shows great promise in controlling the whole-body tumor burden including the metastatic tumor (Slovak et al., 2017).

As one of the thermal ablative approaches, photothermal therapy (PTT) can “burn” tumor with heat generated from the absorbed optical energy by photoabsorbing agents or nanomaterials (Zha et al., 2013; Chen et al., 2016a; Rwei et al., 2017; Yang et al., 2017). The key to complete ablation of the primary tumor is the design of PTT agents that have strong light absorbance, efficient photothermal conversion, excellent biocompatibility, and abundant tumor accumulation (Liu et al., 2015). Various kinds of agents or materials have been reported as effective photothermal agents including carbon nanomaterials (Kim et al., 2015), gold nanoparticles (Ayala-Orozco et al., 2014), organic compounds (Sheng et al., 2014), and indocyanine green (ICG) (Wu et al., 2013; Zheng et al., 2013). Assembly of the supramolecular PTT particles with all FDA-approved materials can promote the clinical translation of photothermal strategy.

Even though ICG is clinically approved for near-infrared (NIR) imagery to identify neoplastic disease during the conduct of surgery, the application of ICG in PTT is limited due to its low photostability and non-tumor-specific distribution *in vivo* (Ma et al., 2013). Free ICG degrades rapidly in aqueous solutions due to the oxidation of double bonds and can be cleared by the liver quickly (*t*_1/2_ ≈ 2–4 min) because of high bonding to plasma proteins (Lajunen et al., 2016). Liposomes, as supramolecular self-assembly nanomaterials, have showed promising results in *in vivo* drug delivery (Elsabahy and Wooley, 2015; Feng et al., 2015, 2016; Ji et al., 2017; Liu et al., 2018; Weldon et al., 2019). Liposomal encapsulation could greatly improve the stability and tumor targeting property of free ICG, while not affecting the PTT property (Ding et al., 2015; Ji and Kohane, 2019). Moreover, both ICG and the composition of liposomes are materials approved by FDA for intravenous (IV) injection, which will greatly guarantee the biocompatibility and future clinical translation (Liu et al., 2013; Zheng et al., 2014). Therefore, we propose a self-assembled ICG-encapsulated liposomal platform for PTT treatment.

Numerous evidences support that the ICG loaded liposome nanoparticles can effectively eradicate the primary tumor, but there are still challenges for inhibiting distant tumors. On one hand, PTT is reported to generate tumor antigens available as an *in-situ* cancer vaccine by destroying tumor cells, inducing systemic anti-tumor response to potentially eliminate metastatic tumors in mouse tumor models (Kostarelos et al., 2009; Guo et al., 2014; Tao et al., 2014) and preliminary clinical trial study (Li et al., 2010), which is called abscopal effect. However, this abscopal effect induced by PTT alone has proven to be weak and not durable (Chen et al., 2016b; Slovak et al., 2017). Recently, researchers have tried to explore the immunological changes after PTT, including inducing enhanced infiltration of CD8^+^ T cells, enhancing the DC maturation, and increasing the secretion of pro-inflammatory cytokines (Chen et al., 2016b; Kleinovink et al., 2017). We deduce that the heat during PTT and overactivation of the immune system after PTT may cause T cell exhaustion-related immune suppression.

T cell exhaustion is a state of T cell dysfunction being well-studied nowadays and is characterized by decreased cytokine production, hypoproliferation, and diminished killing. Cell surface antigen determinants such as program death-1 (PD-1) and T-cell immunoglobulin and mucin domain-containing protein 3 (TIM-3) can be used to identify whether antigen-specific T cells are at an exhaustion stage. Coexpression of different inhibitory receptors has been associated with greater T-cell exhaustion (Fourcade et al., 2010; Wherry and Kurachi, 2015). Reported studies have shown a strong correlation between PD-1 and TIM-3 coexpression, which is a much more severe exhaustion phenotype of CD8 T cells (Sakuishi et al., 2010; Granier et al., 2017). Despite the improvement made on understanding the biologic influence of the expression of PD-1 or TIM-3 on T cell function, the link between PTT treatment and TIM-3 or PD-1 expression in distant tumors has not yet been defined.

**Scheme 1:**
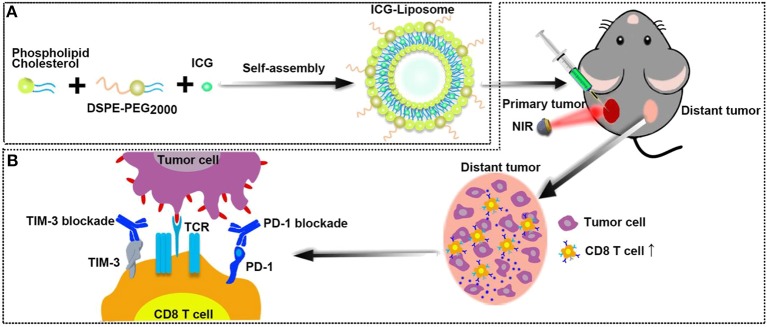
**(A)** The schematic illustration for ICG-loaded liposome as a theranostic nanoplatform. **(B)** The schematic illustration of PTT in the primary tumor and immunological changes in the distant tumor after PTT with dual blockade of PD-1 and TIM-3.

This study aimed to implement a highly efficient and stable supramolecular photothermal system, provide new insights into T cell exhaustion-related immune-suppressive mechanisms limiting PTT efficacy, and explore the potential of immune checkpoint blockade in combination with PTT in cancer therapy. Herein, we optimized phospholipids to formulate biocompatible ICG-liposomes, which were composed of liposome as the encapsulating polymer and ICG as the inner core to enable PTT (Scheme 1A). We injected the ICG-liposome nanoparticles to mice that bear bilateral tumors and applied NIR-induced PTT to primary tumors. Then, we observed the distant tumor growth and characterized PTT induced immune responses as well as PD-1/TIM-3 mediated immune suppression (Scheme 1B). With a comprehensive understanding of the CD8 T cell-related immune microenvironment after PTT, we finally designed a combination therapy with PTT and dual PD-1/TIM-3 blockade for the treatment of distant tumor.

## Materials and Methods

### Materials

Dipalmitoyl phosphatidylcholine (DPPC), phosphoethanolamine-N-[methoxy (polyethylene glycol)-2000] (DSPE-PEG-2000), and cholesterol (CHOL) were obtained from A.V.T. Pharmaceutical Co., Ltd. Company (Shanghai, China). ICG, Non-ionic surfactant Triton X-100 (TX-100), and Cell Counting Kit-8 (CCK-8) were obtained from Sigma-Aldrich. Anti-PD-1 (clone: RMP1-14) and anti-TIM-3 (clone: RMT3-23) used *in vivo* were obtained from Bioxcell. Antibodies against cell surface markers for flow cytometry assay were purchased from Miltenyi and Biolegend. Fetal bovine serum (FBS), DMEM, and RPMI-1640 medium were both obtained from GIBCO Life Technologies Co. Ltd., (USA).

### Preparation of ICG-Liposome Nanoparticles

ICG-loaded liposomes were fabricated using a filming-rehydration method according to the literature (Wang et al., 2014). DPPC, DSPE-PEG-2000, and CHOL were mixed in a round-bottom flask containing 16 ml of chloroform/methanol (5:3) in different ratios (7.2 mg in total). The mixture was dried with a rotary evaporator at 45°C to form a thin film. This film was then hydrated in 5 ml of water with ICG and sonicated for 5 min at 30°C. Furthermore, the obtained solution was sonicated under probe sonication for 30 s. Finally, the prepared ICG-loaded liposome was harvested by removing free ICG through ultrafiltration centrifugation procedure (Millipore, MW 100 kDa).

### Nanoparticle Characterization

The morphology and size of the ICG-liposome were measured by transmission electron microscopy (TEM, JEM-2100F). The hydrodynamic size, polydispersity (PDI), and zeta potential of ICG-liposome were further measured in aqueous solutions by dynamic light scattering (DLS) using Malvern Nano ZS90 (Nano ZS, Malvern, USA).

The absorption spectra of free ICG and ICG-liposome were obtained by a UV-vis spectrophotometer (DU730, Beckman Co., USA) at the wavelength range of 400–1,000 nm. In brief, standard ICG solutions were prepared by diluting ICG with ultrapure water to different concentrations. The prepared solution of ICG-liposome was added to a 5-ml ultrafiltration centrifuge tube (MW 100 kDa), centrifuging for 20 min (4,500 rpm), and finally the subnatant was taken out. The absorbance (Abs) at 810 nm was measured and the ICG concentration in the solution was obtained from the standard curve.

The ICG encapsulation efficiency (EE) and the loading efficiency (LE) were then calculated according to the following equations.

$$\begin{array}{l} \text{EE}\%=\frac{Weight\;of\;ICG\;in\;liposome}{Weight\;of\;ICG\;fed\;initially}\times 100\%\\ \text{LE}\%=\frac{Weight\;of\;ICG\;in\;liposome}{Weight\;of\;liposomes}\times 100\% \end{array}$$

The long-term stability of the ICG-liposome was evaluated in PBS containing 10% fetal bovine serum (FBS) at 37°C by detecting the size distributions with dynamic light scattering (DLS) at various time points.

### *In vitro* Photothermal Efficiency

To measure the photothermal property of ICG-liposome, 4 ml suspensions with different concentrations were placed in a cuvette and irradiated by 808 nm laser (2 W/cm^2^, 10 min), with 4 ml of saline as a negative control. The temperature profile of these suspensions was recorded by a digital thermometer per 10 s.

### Cytotoxicity and *in vitro* PTT Ability Evaluation

MC-38 cells and HEK-293 cells (Human embryonic kidney 293 cells) were cultured in a DMEM medium, containing 10% FBS and 1% penicillin–streptomycin, at 37°C and 5% CO_2_. CT26 cells were cultured in a 1,640 medium containing 10% FBS and 1% penicillin–streptomycin. The culture medium was replaced every other day. The CCK-8 assay was performed on the MC-38 cells, CT26 cells, and HEK-293 cells to investigate the PTT efficiency of ICG-liposome at different concentrations with or without NIR laser irradiation. The cell suspension was dispensed in a 96-well plate, and the plate was preincubated for 24 h in a humidified incubator (at 37°C, 5% CO_2_). Then, the medium was removed and replenished with a fresh medium containing ICG-liposome with different concentrations. After further incubation for 10 h at 37°C, the ICG-liposome suspensions were replaced with a fresh DMEM medium. Besides, the cells in the laser irradiation group were irradiated by 808 nm laser for 5 min at a power density of 2 W/cm^2^. After incubation for another 2 h, the cell viability was measured by CCK-8 assay.

### *In vivo* Antitumor Efficacy of PTT

All animal experiments were carried out in accordance with the Institutional Animal Care and Use Committee (IACUCC) of Sun-Yat Sen University. One hundred microliters of MC-38 tumor cells (1 × 10^6^) or CT-26 tumor cells (1 × 10^6^) suspension was subcutaneously injected in the bilateral flanks of the female C57BL/6 mice or BALB/C mice (6–8 weeks). Mice were divided into groups randomly. The tumor size was measured by a Vernier caliper and calculated according to the following formula: Length × Width × Height × 0.5. PTT was applied when the tumor volume reached about 100 mm^3^.

In order to evaluate the *in vivo* photothermal ablative property of ICG-liposome, six MC-38 tumor-bearing mice were randomly assigned into the saline group (*n* = 3) and the ICG-liposome group (*n* = 3), respectively, and were intratumorally (i.t.) injected with 100 μl of saline or ICG-liposome (400 μg/ml ICG) on the left tumors. The left tumors of these groups were then irradiated with 808 nm laser at 2 W/cm^2^ for 10 min and an IR thermal camera (Ti27, Fluck) was employed to record the region maximum temperature.

To investigate the local PTT effect on distant tumors, 32 MC-38 tumor-bearing mice and 12 CT-26 tumor-bearing mice were randomly divided into the untreated group and the ICG-liposome group, respectively. The left tumors of the ICG-liposome group were treated as described above. After the treatments, the left and right tumor volumes of each mouse were recorded every other day.

### Combinational Therapy of PTT With Dual PD-1 and TIM-3 Blockade

Twenty-five MC-38 tumor-bearing mice were randomly divided into five groups: (1) untreated, (2) PTT only, (3) anti-PD-1 + anti-TIM-3, (4) PTT + anti-PD-1, and (5) PTT + anti-PD-1 + anti-TIM-3. PTT was performed the same as described above. PD-1 and TIM-3 blockades were started 1 day before PTT and were accomplished by administering 200 mg of anti-PD-1 (clone: RMP1-14, BioXCell) or anti-TIM-3 (clone: RMT3-23, BioXCell) through i.p. injection to mice every 3 days for a total of three times.

### Flow Cytometry Analysis

To study the immune cells in distant tumors, right tumors were harvested from mice in different groups (*n* = 5) and stained with Viobility 405/520 Fixable Dye (Miltenyi), CD45.2 APC-CY7 (Biolegend, Clone: 104), CD3e FITC (Biolegend, Clone: 17A2), CD8a PE-vio615 (Miltenyi, Clone: REA601), PD-1 PE (Biolegend, Clone: 29F.1A12), TIM3 APC (Miltenyi, Clone: REA602), CD4 VioBlue (Miltenyi, Clone: REA604), and Foxp3 Alexa Fluor 700(Biolegend, Clone: MF-14.1A12) antibodies, according to the manufacturer's protocols.

Briefly, tumor tissues were cut into small pieces and digested with collagenase and DNase. Then, cell suspension was filtered through a 75-μm cell mesh and resuspended in PBS (pH 7.4) with 0.5% FBS for further analysis. Flow cytometric analysis was performed using a FACS LSRFortessa flow cytometer (BD).

Tumor-infiltrating cytotoxic T lymphocytes (CTL) and helper T cells were CD45^+^CD3^+^CD4^−^CD8^+^ and CD45^+^CD3^+^CD4^+^CD8^−^, respectively. Then, the expressions of PD-1 and TIM-3 in cytotoxic T lymphocytes were analyzed. Further, CD4^+^ helper T cells were classified into regulatory T cells (Tregs) (CD3^+^CD4^+^Foxp3^+^) and effective T cells (CD3^+^CD4^+^Foxp3^−^).

### Immunofluorescence

Tumor tissue samples obtained were frozen in OCT immediately and stored at −80°C. Frozen samples were sectioned at 5 mm with a cryostat, placed on slides, and air-dried. Then, the slides were fixed for 5 min with 100% acetone, pretreated with avidin/biotin blocker (DAKO) for 10 min, and blocked with 5% donkey serum (DAKO) in PBST for 30 min. Staining for CD8 (Biolegend, Clone: 53-6.7) was performed using non-labeled primary antibodies followed by Cy3 fluorophore-labeled secondary antibodies. Nuclei were then highlighted with a DAPI mounting medium.

### Statistical Analysis

Statistical analyses were performed using Prism 8 software (GraphPad Software Inc.). Data from animal experiments were expressed as mean ± SEM or mean ± SD for biologic replicates. Statistical significance was calculated by Student's *t*-test. Statistical significance for survival analysis was calculated by the log-rank test (^***^P < 0.001, ^**^P < 0.01, ^*^P < 0.05).

## Results and Discussion

### Supramolecular ICG-Liposomes Formulation and Characterization

We first synthesized and optimized the formulation of ICG-loaded liposomes. DPPC, DSPE-PEG-2000, and cholesterol were used to encapsulate the NIR dye ICG by a filming-rehydration method, wherein the mixed lipids were employed to form the liposome loaded with ICG. Different weight ratios of lipid compositions (DPPC/CHOL/DSPE-PEG2000) with different ICG/lipid weight ratios were prepared and their sizes as well as ICG loading efficiency were analyzed (Table S1). The final ICG-loaded liposomes had a mean diameter from 82.8 to 165.9 nm, and low polydispersity indexes (PdI) from 0.194 to 0.349, indicating a relatively uniform size distribution, which is consistent with the TEM result (Figure S1). The high ICG/lipid ratio (1:5) reduced the encapsulation efficiency of ICG in the liposomes, compared to the relative lower ICG/lipid ratio (1:10). Formulation F with a lipid compositions ratio of 6:3:1 and an ICG/lipid ratio of 1:10 had the highest ICG loading efficiency (Table S1). Thus, the formulation F was used in further experiments.

**Figure 1:**
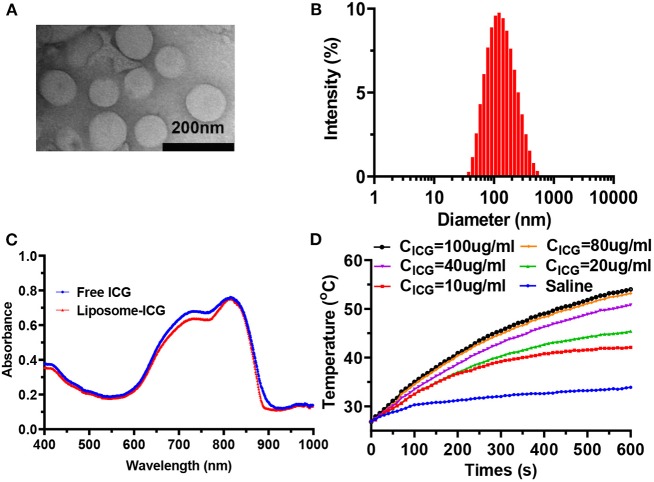
**(A)** TEM image of ICG-liposome. **(B)** Hydrodynamic diameters of ICG-liposome measured by DLS. **(C)** UV-vis-NIR spectra of ICG-liposome and free ICG, indicating the successful loading of ICG into hybrid liposome. **(D)** Temperature curves of saline and ICG-liposome at various concentrations under the 808 nm light irradiation (2 W/cm^2^, 10 min).

In the transmission electron microscope (TEM) image, the obtained supramolecular nanoparticles (Formulation F) showed homogenous sizes and a well-defined spherical shape (Figure 1A). The average hydrodynamic size of nanoparticles (Figure 1B) was 121.4 ± 1.2 nm, with a PDI of 0.254 ± 0.008, measured by the DLS, which is consistent with the TEM result. Moreover, the zeta potential of liposome was measured to be −6.4 ± 0.5 mV in water. The characteristic absorption peak of ICG was found in the ultraviolet-visible-NIR absorption spectrum of nanoparticles, showing the successful encapsulation of ICG in the core (Figure 1C). The ICG-liposome exhibited remarkable absorption at 810 nm in the UV-vis spectrum, which is suitable for biological application. The encapsulation efficiency of ICG was 98.6% and the ICG loading efficiency in the liposome was 9.1%, as measured by the UV-vis spectroscopy and calculated through the ICG standard curve (Figure S2).

The photothermal properties of ICG-liposome were then investigated under 808 nm laser irradiation (Figure 1D, Figure S3). Significant temperature elevation was observed when the ICG-liposome suspension was irradiated by an 808 nm laser (2 W/cm^2^ for 10 min), while saline solution showed no obvious temperature increase under the same condition, indicating that the NIR laser is safe for normal tissue. TEM imaging of ICG-liposome showed that a substantial number of liposome microbubbles were observed after 808 nm laser irradiation, which was consistent with the DLS result (Figures S4A,B). Moreover, the ICG-liposome showed an ICG concentration-dependent temperature increment. The temperature of ICG-liposome solution (40 μg/ml) notably increased about 23.9°C at 2 W/cm^2^ after irradiation for 10 min, with no obvious temperature elevation in saline. All these results show that the ICG-liposome has an efficient photothermal conversion ability.

### ICG-Liposome Showed Favorable Photothermal Abilities Both *in vitro* and *in vivo*

**Figure 2:**
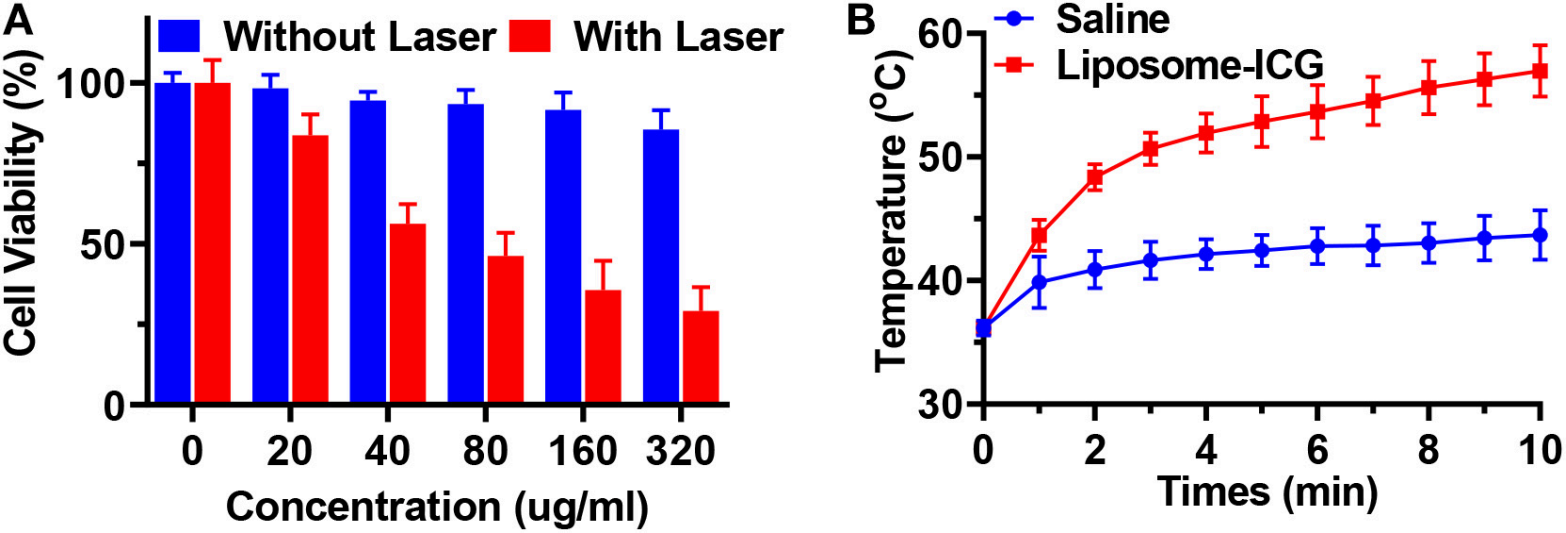
**(A)** The cell-killing effect of MC-38 cells incubated with ICG-liposome of different concentration with or without NIR laser irradiation (808 nm, 2 W/cm^2^, 10 min, *n* = 4). **(B)** Temperature elevations of MC-38 tumor-bearing mice (*n* = 3). Data are presented as means ± SD.

ICG-liposome showed remarkable stability in 10% FBS-containing PBS over 96 h, with nearly unchanged size (Figure S5). The cytotoxicity and enhanced photothermal therapeutic effect of ICG-liposome at the cellular level were evaluated by CCK-8 assay of MC-38 cells, CT26 cells, and HEK-293 cells (Figure 2A). No obvious cytotoxicity of ICG-liposome was found at the concentration of 320 μg/ml without laser irradiation in MC-38 cells, with a cell viability of 85.5%, suggesting good biocompatibility. As a contrast, the viabilities of MC-38 cells receiving ICG-liposome + laser treatment decreased dramatically to 29.18%, much lower than that of MC-38 cells (85.5%) treated with ICG-liposome only. Similar results were also found in CT26 cells and HEK-293 cells (Figure S6).

The PTT effect of ICG-liposome was studied in MC-38 tumor-bearing C57BL/6 mice, compared with saline (Figure 2B). When the tumors reached approximately 100 mm^3^, 100 μl of ICG-liposome or saline was intratumorally (i.t.) injected. After being irradiated by 808 nm laser for 10 min, the temperature of the tumor in the saline group increases slightly to 43.7°C. However, the temperature of the tumor in the ICG-liposome group was 57.0°C, which was much higher than that in the saline group. The mice treated with the ICG-liposome + NIR group presented obvious tumor growth inhibition, and tumors were fully cleared about 3 days later with black scars left. These results indicated that the encapsulated ICG could efficiently ablate tumors *in vivo* by converting the NIR light into heat. Taken together, ICG-liposome may be applied as an efficient PTT-enhancing agent for tumor ablation.

### Local PTT Induced Effective but Short-Lived Growth Inhibition of Distant Tumors

**Figure 3:**
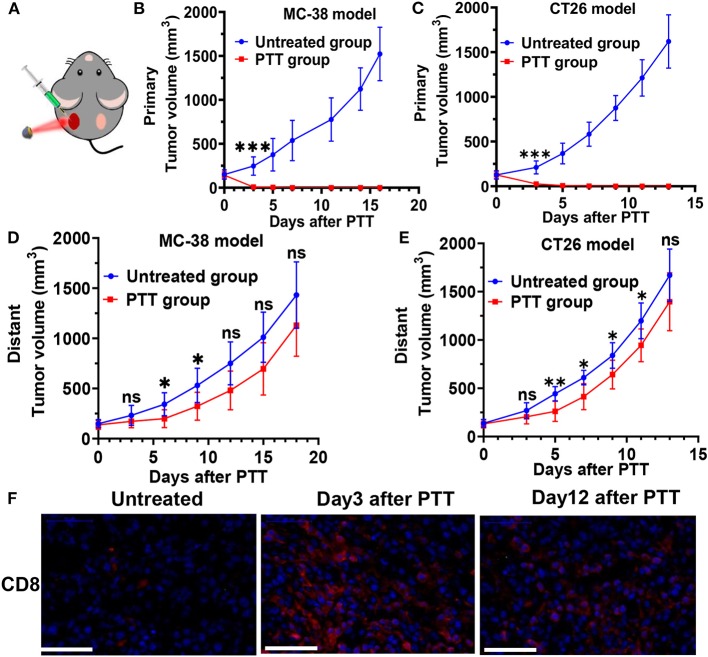
**(A)** Schematic illustration of ICG-liposome-based PTT on mice. Primary tumor growth profile of MC-38-bearing **(B)** and CT26-bearing **(C)** mice from each group. Distant tumor outgrowth curves of double MC-38 tumor-bearing mice **(D)** and double CT-26 tumor-bearing mice **(E)** in which the primary tumor was left untreated or PTT-treated. **(F)** CD8 T cells infiltration of each group in the MC-38 tumor microenvironment on day 3 and 12 after treatment was examined by immunofluorescence. *n* = 6 in each group. Data are presented as means ± SD. Statistical significance was calculated by Student's *t*-test: ***P < 0.001, **P < 0.01, *P < 0.05.

In further *in vivo* experiment, BALB/c mice and C57BL/6 mice were inoculated with CT26 and MC-38 on bilateral flanks, respectively. PTT was then performed on the tumor at the left flank once tumors reached approximately 100 mm^3^. BALB/c mice bearing CT26 tumors and C57BL/6 mice bearing MC-38 tumors in the PTT treatment group were intratumorally (i.t.) injected with ICG-liposome on the left tumor. Then, the left tumors of treatment groups were irradiated with 808 nm laser at 2 W/cm^2^ for 10 min (Figure 3A). The left tumor temperature of mice injected with ICG-liposome under laser irradiation quickly rose to nearly 60°C, as monitored by an infrared thermal camera (Figure 2B). After the treatments, the right tumor volume of each mouse was recorded every other day. PTT-treated left tumors in two tumor-bearing mice regressed and were soon cleared (Figures 3B,C). On day 3, the left treated tumors almost disappeared with only black scars left.

Furthermore, in both the CT26 and MC-38 tumor model, we found that untreated distant tumors grew significantly slower at the early stage, if the contralateral tumors received PTT treatment (Figures 3D,E). In the MC-38 tumor model, the average distant tumor volume in the PTT group was 199.0 mm^3^, much smaller than that of the untreated group (342.6 mm^3^) on day 6 after PTT. Similarly, the average distant tumor volume in the PTT group of CT-26 tumors was 41% smaller than that of the untreated group on day 5. These systemic antitumor effects by local ablative therapy are also known as the abscopal effect and have been shown in many localized ablation therapies (Formenti and Demaria, 2009; Bastianpillai et al., 2015). However, on around days 6–8, the distant tumor restored its progressive growth and there were no significant difference (*P* > 0.05) of tumor volume between the PTT group and the untreated group at the final stage in both MC-38 and CT-26 tumor models.

### Enhanced Tumor-Infiltrating T Cells Played an Important Role in Distant Tumor Growth Inhibition

**Figure 4:**
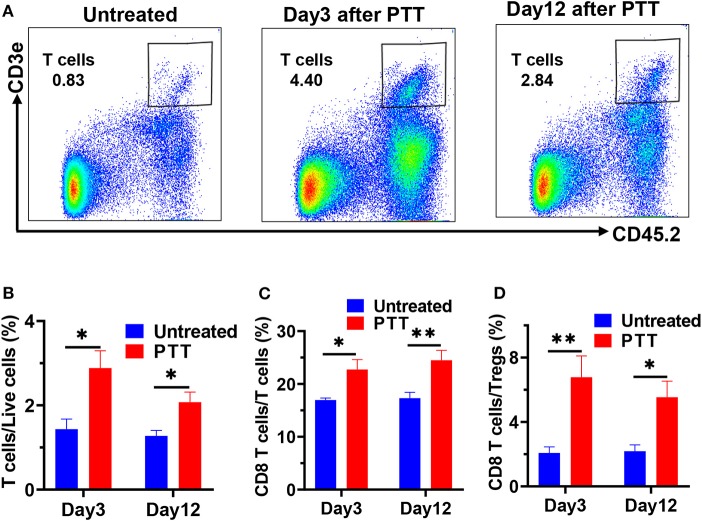
**(A)** Representative flow cytometric plots showing CD45^+^CD3^+^ T cells in single live cell suspension of MC-38 tumors on days 3 and 8 after PTT. Flow cytometry results showing the percentage of CD45^+^CD3^+^ T cells **(B)**, CD8^+^ tumor-infiltrating T cells **(C)**, and the ratio of CD8 T cells to Tregs **(D)** on days 3 and 12 after PTT in distant MC-38 tumors. *n* = 5 in each group. Data are presented as means ± SEM. Statistical significance was calculated by Student's *t*-test: **P < 0.01, *P < 0.05.

To further understand the dynamic changes and mechanisms of antitumor immune responses induced by PTT, we conducted an immune analysis of the distant MC-38 tumor at an earlier and later time point after PTT treatment. In flow cytometry analyses, we digested the distant tumors into single-cell suspension and used CD45 as well as CD3 as the markers to gate tumor-infiltrating T cells (Figure 4A). Then, CD8 and CD4 markers were used to further gate out the CD8 T cells and CD4 T cells (Figure S7). Furthermore, we analyzed the PD-1 and TIM-3 expression on CD8 T cells, which indicated the exhaustion of CD8 T cells. Besides, CD4 T cells could be further classified into immune active effective T cells (CD4^+^Foxp3^−^) and regulatory T cells (Tregs) (CD4^+^Foxp3^+^), which could inhibit antitumor immune responses.

As a result, a roughly two-fold increase in the percentage of CD45^+^CD3^+^ T cells was observed in the right tumor of PTT-treated mice (Figure 4B) on day 3 after PTT treatment, compared with the untreated group. This increase in T cells can still be found on day 12, which indicates that primary tumor PTT therapy induced a durable immune response in the distant tumors even when the tumor regained rapid growth at a later stage.

Furthermore, increased frequencies of CD8^+^ T cells contributed the most to the T cell infiltration (Figures 3F, 4C), as CD4^+^ T cells only slightly increased on day 3 and had no significant difference with the untreated group on day 12 (Figure S8). Interestingly, the percentage of CD8 T cells further increased to 24.48% on day 12, revealed by immunofluorescence staining and flow cytometry (Figures 3E, 4C), suggesting that CD8^+^ T cells may directly mediate the abscopal effect of local PTT. Besides, increase in the CD8/Tregs ratio (3.26-fold increase; Figure 4D) indicates an enhanced antitumor immunity on day 3. However, on day 12, this increase in the CD8/Tregs ratio became less significant (2.81-fold increase), as Tregs of the PTT-treated mice increased to a similar level of the untreated ones.

This effect is likely caused by enhanced cross-presentation of tumor antigens by dendritic cells, as well as the immunostimulatory effects of PTT-induced cell death (Mroz et al., 2011). This observation is consistent with the reported results (Kleinovink et al., 2017; Chen et al., 2019). Intracellular tumor-specific antigens are released during the thermal ablation reduced tumor destruction and captured by antigen-presenting cells (APCs). Moreover, the hyperthermic temperature can also fire up adaptive immunity and increase the ability of APCs to sense ongoing tumor antigens, to present these antigens to T cells (Knippertz et al., 2011). Published data show that exposure of dendritic cells to hyperthermic temperatures (39.5–41.5°C) *in vitro* could upregulate their expression of MHC class II, TLR4, and the co-stimulatory molecules CD80 and CD86 (Ostberg et al., 2003; Yan et al., 2007). Once mature antigen-loaded dendritic cells reached lymph nodes, T cells are activated and expanded. Exposure of tumor-bearing mice to hyperthermic temperatures was also found to increase the trafficking of activated CD8 T cells to tumor sites (Evans et al., 2015).

Thus, all these results provide evidence that local PTT treatment triggers a systemic CD8 T cell-dependent anti-tumor effect on untreated distant tumors.

### Restored Progressive Distant Tumor Growth Later After PTT Was Correlated to Compensatory Upregulation of PD-1 and TIM-3 in Tumor-Infiltrating CD8 T Cells

**Figure 5:**
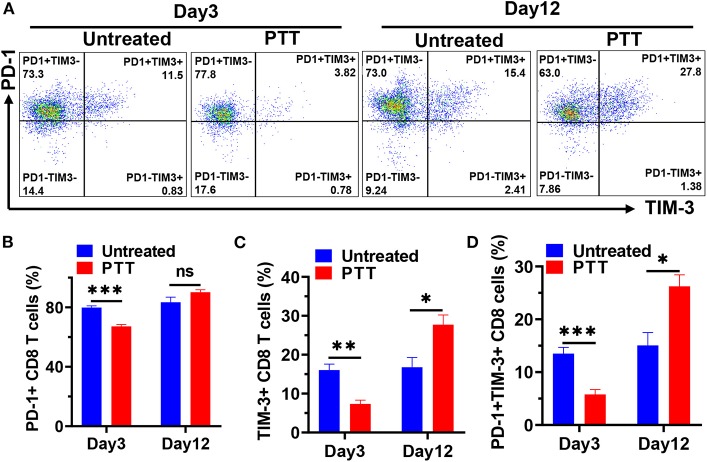
**(A)** Representative flow cytometric plots showing PD-1 and TIM-3 expression in tumor-infiltrating CD8 T cells on days 3 and 12 after PTT. Flow cytometry results showing the percentage of PD-1^+^ **(B)**, TIM-3^+^ **(C)**, and PD-1^+^TIM-3^+^ **(D)** CD8 T cells on days 3 and 12. *n* = 5 in each group. Data are presented as means ± SEM. Statistical significance was calculated by Student's *t*-test: ***P < 0.001, **P < 0.01, *P < 0.05.

Local PTT treatment inhibited the growth of distant tumors via increased CD8^+^ T cell infiltration but failed to fully clear them, resulting in the rapid tumor progression at a later stage. To understand which pathways may drive immune suppression and limit T cell activity, we analyzed the typical exhaustion markers to explore whether the increased CD8 T cells became exhausted after PTT treatment. Here, we tried to examine the PD-1 and TIM-3 expression of CD8 T cells in the TME of distant MC-38 tumors after PTT therapy (Figure 5A). Our flow cytometry data showed that nearly 79.9% of CD8 T cells expressed PD-1 and about 16.1% of CD8 T cells expressed TIM-3 in MC-38 tumors from untreated mice (Figures 5B,C). On day 3, we observed a rapid downregulation of both PD-1 and TIM-3 expression on tumor-infiltrating CD8 T cells of the PTT group. The percentage of PD-1^+^ CD8 T cells decreased to about 67.2% (*p* < 0.001), while TIM-3 expression decreased to nearly 7.4% (*p* < 0.01) (Figures 5B,C). Interestingly, the percentage of cells coexpressing PD-1 and TIM-3 was also significantly decreased from about 13.5% to 5.8% (*p* < 0.001; Figure 5D). These results indicated that CD8 T cells expressed less exhausted markers and had better function in the early time after PTT, consistent with the efficient inhibition of tumor growth. However, the frequency of PD-1^+^ CD8 T cells of PTT-treated mice increased to a similar level of untreated ones on day 12. Meanwhile, TIM-3 on CD8 T cells was elevated to 27.7% on day 12 and was significantly higher than that in the untreated mice (*p* < 0.05). Besides, on day 12, PD-1^+^TIM^+^ CD8 T cells, which represented the most exhausted type, were observed to increase five-fold than that on day 3 and two-fold than untreated ones (Figure 5D).

In this study, we show for the first time in PTT-treated tumor-bearing mice that PD-1 and TIM-3 coexpression in tumor-infiltrating CD8 T cells predominantly increased at the later stage of the distant tumor progression. These PD-1 and TIM-3 double-positive CD8 T cells were highly exhausted and had markedly reduced cytokine production as reported in patients with metastatic melanoma (Fourcade et al., 2010). The deceased PD-1 and TIM-3 expression in the early stage may be caused by the fact that a new subset of CD8 T cells expressing low levels of PD-1 and TIM-3 were activated in the peripheral immune organ and trafficked to distant tumors. In the later stage, these newly trafficked CD8 T cells were exposed to high levels of tumor antigens and reformed in the tumor microenvironment to become exhausted.

Collectively, these results showed that the expression of both PD-1 and TIM-3 underwent dynamic changes after PTT treatment, with significant decrease at the early time point and dramatic increase at a later stage. The data indicating that tumor-infiltrating CD8 T cells underwent functional exhaustion by compensatory upregulation of checkpoint receptors may explain the rapid regression later after PTT.

### Dual Blocking PD-1 and TIM-3 in the PTT-Treated Mice Induces Robust Systemic Antitumor Immunity to Inhibit the Growth of Distant Tumors

**Figure 6:**
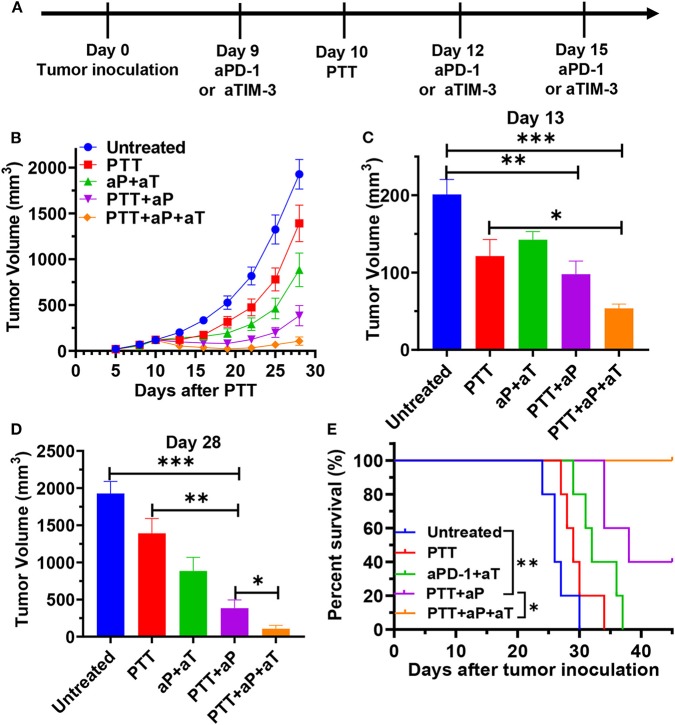
**(A)** Schematic illustration of ICG-liposome-based PTT and dual blocking PD-1 and Tim-3 combination therapy to inhibit distal tumor growth. MC-38-bearing mice were then treated with PTT, anti-PD-1 + anti-TIM-3, PTT + anti-PD-1, and PTT + anti-PD-1 + anti-TIM-3. Tumor growth **(B)** and Kaplan–Meier survival curves **(E)** of tumor-bearing mice are shown. Pooled data of day 13 **(C)** and day 28 **(D)** from the experiments shown in **(B)**. aP and aT represent anti-PD-1 and anti-TIM-3 therapy, respectively. *n* = 5 in each group. Data are presented as means ± SEM. Statistical significance in **(B–D)** was calculated by Student's *t*-test. Statistical significance for survival analysis in (E) was calculated by the log-rank test. ***P < 0.001, **P < 0.01, *P < 0.05.

The results described above provide a basis for testing rational combination treatments of immune checkpoint blockade with PTT treatment. Therefore, we analyzed whether enhancing the PPT-induced CD8 T cell response by PD-1 or TIM-3 blockade would enable enhanced tumor eradication. We treated double MC38 tumor-bearing mice with PTT of left tumors and conducted systemic PD-1 blockade or dual blocking PD-1 and TIM-3 during the treatment phase. MC-38 bearing mice were treated with PTT alone, PTT plus anti-PD-1 antibodies (PTT + aPD-1), PTT plus anti-PD-1, and anti-TIM-3 antibodies (PTT + aPD-1 + aTIM-3), anti-PD-1 plus anti-TIM-3 antibodies (aPD-1 + aTIM-3), or left without treatment (Figure 6A). As a result, we found that PTT alone modestly inhibited distant tumor growth at an early stage, consistent with our previous data (Figures 6B,C). Dual blocking PD-1 and TIM-3 themselves also modestly delayed tumor growth, as compared with the untreated group, similar to the reported result (Sakuishi et al., 2010). However, both PTT treatment alone and immune checkpoint blockade alone were not efficient enough to fully control the distant tumors. In contrast, combination treatment with PPT and PD-1 blockade combined the strong respective effects of each treatment and exhibited superior tumor regression (with an average tumor volume 72.4% smaller than the PTT group on day 28), longer tumor inhibition duration, and prolonged survival (*p* < 0.0001) compared to the PTT treatment alone (Figures 6D,E).

Additional blockade of the TIM-3 pathway further enhanced the synergistic effect of PTT and blocking PD-1 on antitumor immunity in the MC-38 tumor-bearing mice and had an additive effect in delaying distant tumor growth (277.6 mm^3^ smaller on day 28) and prolonging survival time (*p* < 0.05) as compared with the PTT + aPD-1 group (Figures 6D,E). Above all, PTT combined with dual antibody blockade against PD-1/TIM-3 resulted in tumor-free survival in 40% of treated mice, compared to that combination with single PD-1 blockade that led to tumor outgrowth in all animals. This advanced synergistic effect may be due to the further upregulation of TIM-3 resulting from single blockade of PD-1, which has been reported (Huang et al., 2017). Single blockade of PD-1 can lead to a compensatory induction of TIM-3, LAG-3, and CTLA-4 and other checkpoint inhibitors, which builds a feedback loop that acts to mediate local immune suppression (Huang et al., 2017). Based on the reported data, we hypothesized that compensatory and/or overlapping functionality for PD-1 and TIM-3 may limit the efficacy of a single checkpoint blockade. PTT combined with a dual block of PD-1 and TIM-3 would lead to the best distant tumor control.

Taken together, these results suggest that the combinatorial blockade of PD-1 and TIM-3 has significant benefit in controlling distant tumor growth when the primary tumor is completely ablated by PTT. These data provide a basis for PTT with combinatorial checkpoint blockade in clinical intervention for tumor therapy.

## Conclusions

In summary, an ICG-loaded supramolecular theranostic nanomedicine ICG-liposome was synthesized to achieve NIR triggered photothermal ablation. Compared to the free compound, packing ICG with optimized phospholipids in a self-assembly way exhibited enhanced water solubility and superior stability and obtained high encapsulation yields (>95%), as well as excellent NIR light-triggered PTT ability. After irradiation, the encapsulated ICG can efficiently transfer NIR light into tumor-ablating heat. We find that PTT of mouse colon tumors mediated strong primary tumor ablation and eradication, which also delayed distant tumor growth at the early time by increasing tumor infiltrating T cells. Our data further reveal that restored progressive distant tumor growth later after PTT may be caused by compensatory upregulation of PD-1 and TIM-3 in tumor-infiltrating CD8 T cells, limiting T cell antitumor efficacy. This compensatory upregulation of immune checkpoints also provides an opportunity for choosing appropriate blockade agent(s) for combinatorial treatment. Furthermore, using a mouse colon cancer model, we observed that a combination of localized PTT and anti-PD-1 antibodies significantly enhanced distant tumor growth inhibition and prolonged survival. Interestingly, additional blockade of the TIM-3 pathway further enhanced the synergistic effect. The efficacy of photodynamic therapy and all these synergistic effects of supramolecular ICG-loaded liposome prove it to be a good antitumor agent.

## Data Availability Statement

All datasets generated for this study are included in the article/Supplementary Material.

## Ethics Statement

The animal study was reviewed and approved by ICE for Clinical Research and Animal Trials of the First Affiliated Hospital of Sun Yat-sen University.

## Author Contributions

T-YH, MX, MK, and X-YX conceived and designed the experiments and also analyzed the data. C-YZ synthesized the liposomes. G-LH, B-WZ, B-XL, J-YY, and L-YS performed animal experiments. The manuscript was prepared by T-YH and edited by MX. All authors have approved the final version of the manuscript.

### Conflict of Interest

The authors declare that the research was conducted in the absence of any commercial or financial relationships that could be construed as a potential conflict of interest.
