# Supplementary material for: Supramolecular Photothermal Nanomedicine Mediated Distant Tumor Inhibition via PD-1 and TIM-3 Blockage
Source: Front Chem. 2020 Feb 14;8:1. doi: 10.3389/fchem.2020.00001 (PMC7034522; doi:10.3389/fchem.2020.00001)
Supplement: Supplementary file 1 [file Data_Sheet_1.pdf]

Supplementary Material

Table 1. Optimization of ICG-Liposome with different formulations. N=3 in each group. Data are presented as means ± SD.

Formulations	Weight ratio of (DPPC / CHOL/ DSPE-PEG)	ICG/lipid weight ratio	Liposome diameter (nm ± SD)	PdI	ICG loading efficiency (%)
A	5:3:1	1:5	154.4 ± 2.6	0.349 ± 0.047	7.6 ± 0.4
B	5:3:1	1:10	165.9 ± 2.8	0.275 ± 0.004	6.7 ± 0.2
C	6:2:1	1:5	82.8 ± 1.9	0.228 ± 0.009	8.6 ± 0.4
D	6:2:1	1:10	136.6 ± 0.8	0.332 ± 0.005	8.0 ± 0.4
E	6:3:1	1:5	90.7 ± 3.2	0.194 ± 0.018	8.2 ± 0.3
F	6:3:1	1:10	121.4 ± 1.2	0.254 ± 0.008	9.1 ± 0.2

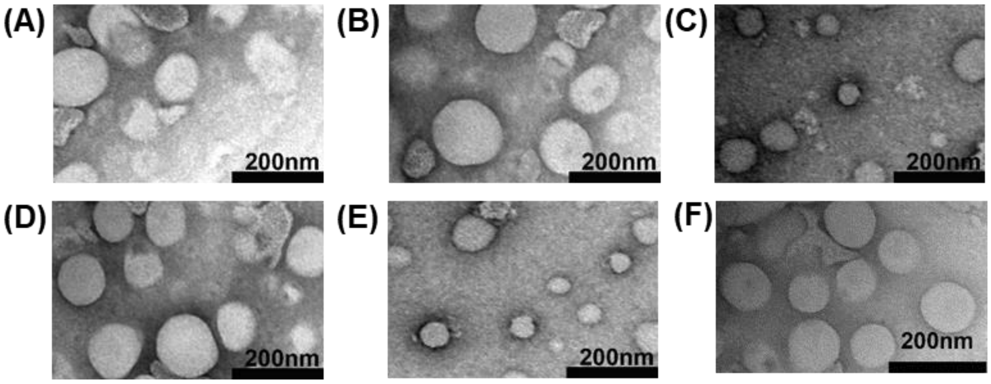

Supplementary Figure 1. TEM image of Liposome-ICG Formulation A **(A)**, B **(B)**, C **(C)**, D **(D)**, E **(E)**, and F **(F)**.

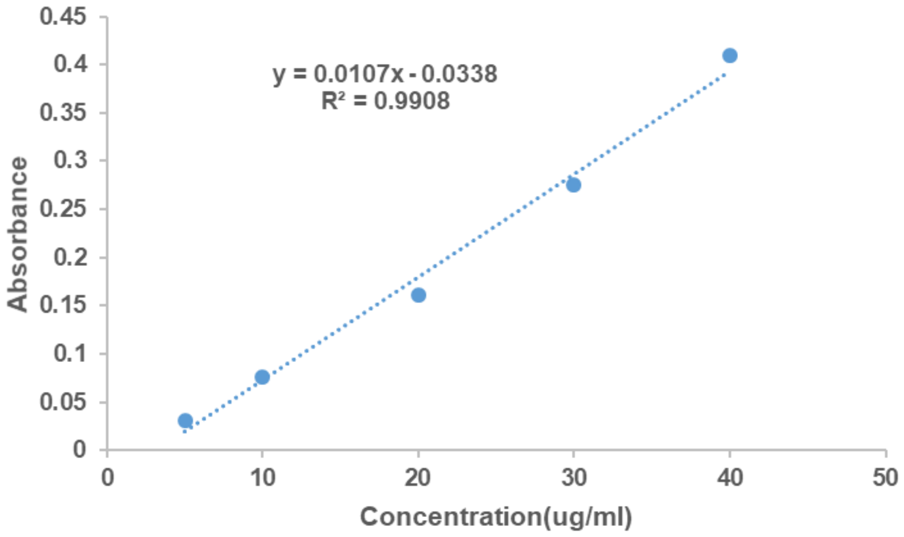

Supplementary Figure 2. The absorbance at 810 nm was measured on a UV-Vis/NIR spectrophotometer and standard curve of ICG concentration was obtained.

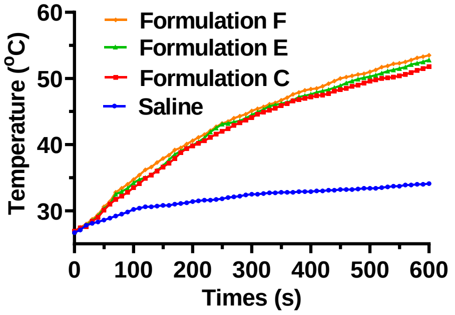

Supplementary Figure 3. Temperature curves of Saline, Formulation C, E and F (ICG-Liposome at same ICG concentrations of 80 µg/mL) under the 808 nm light irradiation (2 W/cm^2, 10 min).

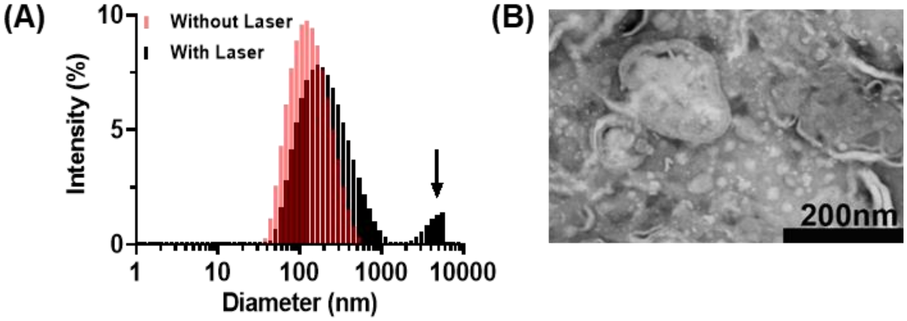

Supplementary Figure 4. (A) Hydrodynamic diameter of Formulation F without or with laser irradiation (808 nm 2W/cm^2, 10 min). **(B)** TEM image of Formulation F with laser irradiation (808 nm 2W/cm^2, 10 min).

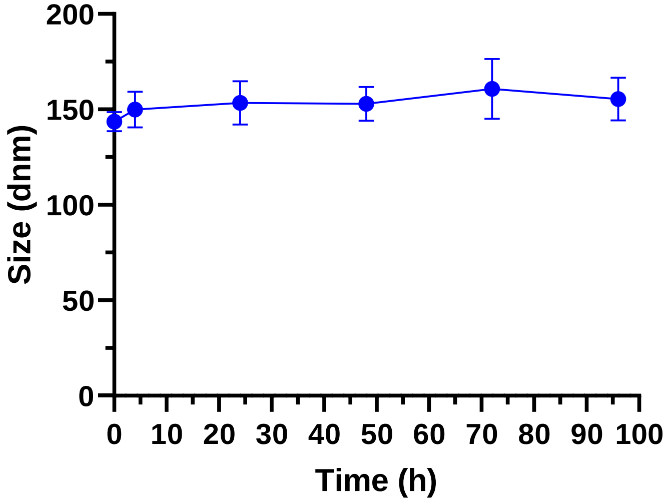

Supplementary Figure 5. Stability of the Liposome-ICG incubated in PBS with 10% FBS; data are represented as mean ±SD (n= 3).

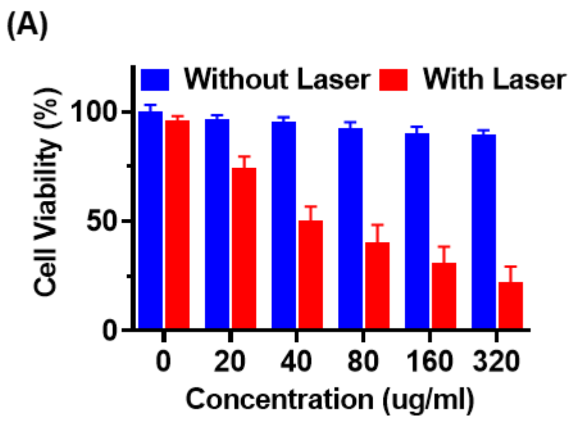

Supplementary Figure 6. The cell-killing effect of CT-26 cells **(A)** and HEK-293 cells **(B)** incubated with Liposome-ICG of different concentration with or without NIR laser irradiation (808 nm, 2 W/cm^2, 10 min, n =4).

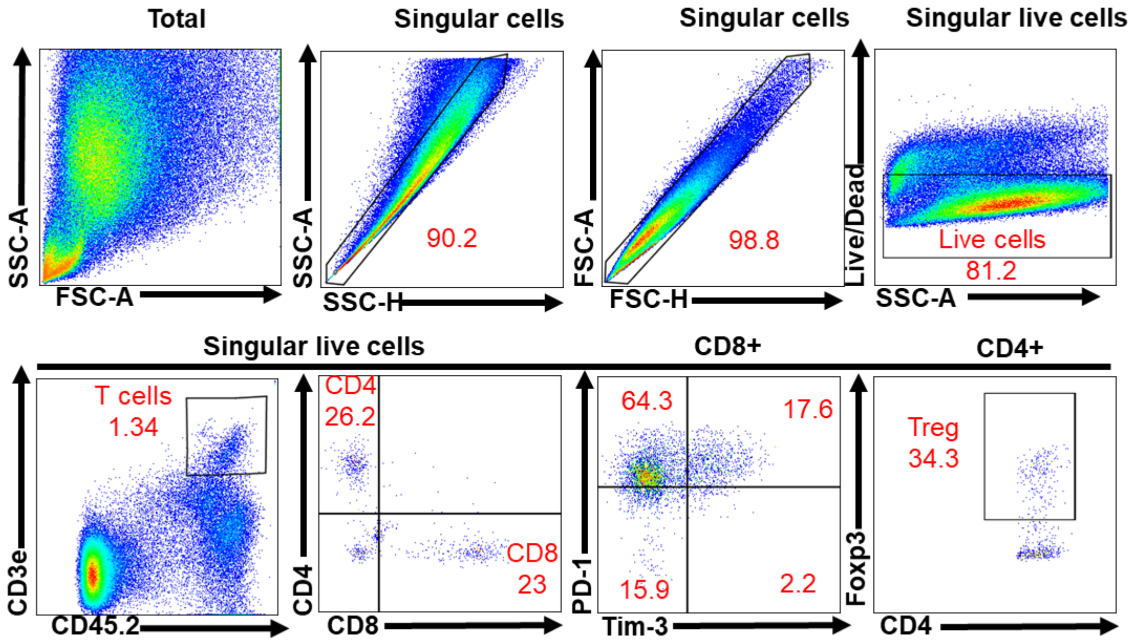

Supplementary Figure 7. Gating strategy for flow cytometry analysis of living singular cells(Live/dead$^-$), T cells (CD45.2$^+$CD3ε$^+$), CD4 T cells (CD4$^+$), CD8 T cells (CD8$^+$), PD-1 and Time-3 expression in CD8 T cells , and Tregs (CD4$^+$Foxp3$^+$) in MC-38 tumor from C57BL/6 mice.

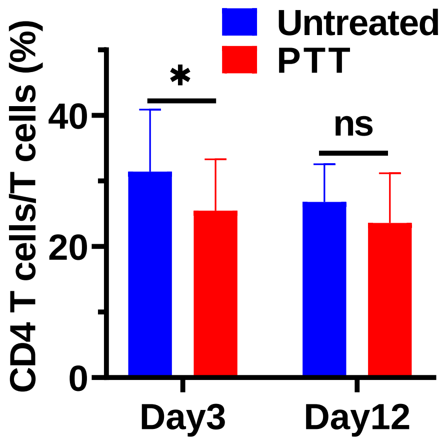

Supplementary Figure 8. Flow cytometry results showing the percentage of $CD4^{+}$ T cells on days 3 and 12 after PTT in distal MC-38 tumors. N=5 in each group. Data are presented as means ± SEM. Statistical significance was calculated by Student's t-test: *** P < 0.001, **P < 0.01, *P < 0.05.
